# Periesophageal Pseudoaneurysms: Rare Cause of Refractory Bleeding Treated with Transarterial Embolization

**DOI:** 10.1155/2016/1456949

**Published:** 2016-10-12

**Authors:** Divyanshoo R. Kohli, Rachit D. Shah, Daniel J. Komorowski, George B. Smallfield

**Affiliations:** ^1^Division of Gastroenterology and Hepatology, Virginia Commonwealth University, Richmond, VA, USA; ^2^Division of Cardiothoracic Surgery, Virginia Commonwealth University, Richmond, VA, USA; ^3^Division of Vascular and Interventional Radiology, Virginia Commonwealth University, Richmond, VA, USA

## Abstract

A 43-year-old female with history of systemic lupus erythematosus, prior cytomegalovirus esophagitis treated with ganciclovir, and long segment Barrett's esophagus (Prague class C8 M9) with high grade dysplasia treated with radiofrequency ablation presented to the hospital with hematemesis. An upper gastrointestinal endoscopy showed multiple esophageal ulcers with active arterial spurting which could not be controlled with endoscopic interventions including placement of hemostatic clips. An emergent angiogram demonstrated actively bleeding saccular dilations (pseudoaneurysms) in the esophageal branches of the lower thoracic aorta as well as left gastric artery for which gelfoam and coil embolization was initially successful. Due to recurrence of massive bleeding, she subsequently underwent emergent esophagectomy and bipolar exclusion. Pathology demonstrated submucosal hemorrhage, esophagitis with dysplastic Barrett's mucosa, and an ulcer containing cytomegaloviral inclusions. We report the first case of arterial bleeding from periesophageal pseudoaneurysms as well as use of angiographic embolization for arterial bleeding in the esophagus.

## 1. Case

A 43-year-old female presented to the hospital with hematemesis and hemorrhagic shock. She had a medical history of systemic lupus erythematosus (SLE), prior cytomegalovirus esophagitis treated with ganciclovir (DNA negative after therapy), and long segment Barrett's esophagus (Prague class C8 M9) with high grade dysplasia treated with radiofrequency ablation in the past.

**Figure 1:**
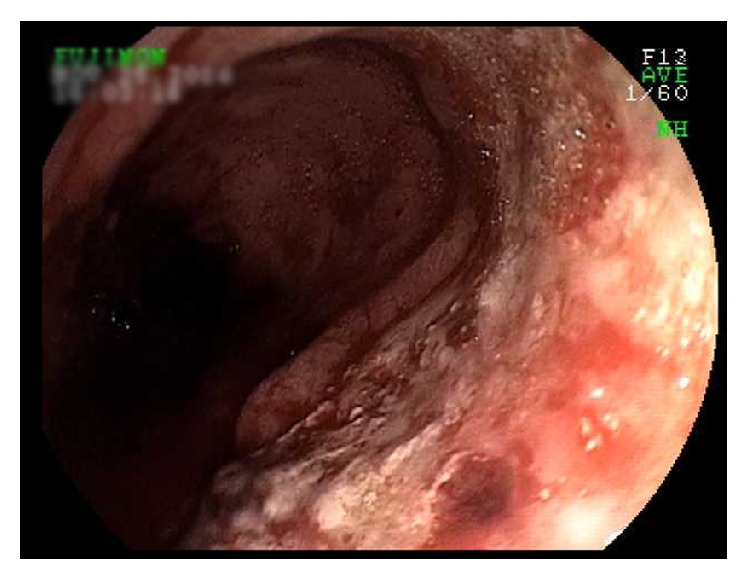
Esophageal ulcers on upper endoscopy.

**Figure 2:**
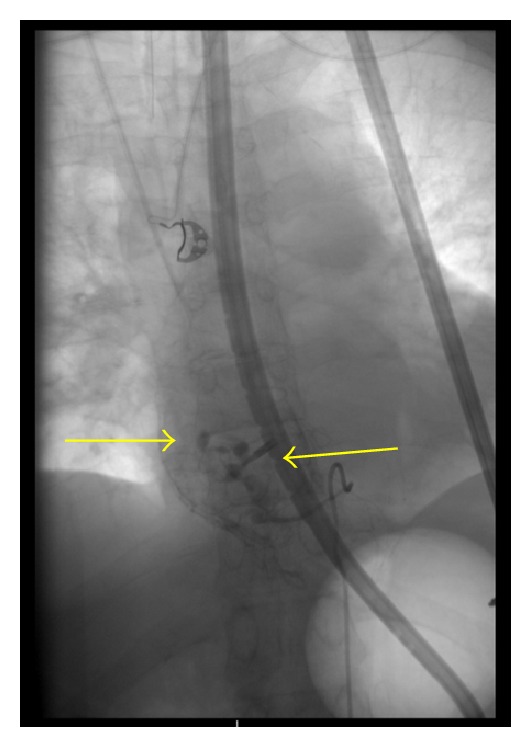
Angiography showing saccular dilations (pseudoaneurysms) and a clip (arrows).

The patient was resuscitated with fluids and blood products. An upper gastrointestinal endoscopy showed multiple esophageal ulcers with active arterial spurting which could not be controlled with endoscopic interventions including placement of hemostatic clips (Figure 1). An emergent angiogram demonstrated actively bleeding saccular dilations (pseudoaneurysms) in the esophageal branches of the lower thoracic aorta which supply the lower esophagus. Embolization of the arteriole with gelfoam and coils was successfully performed (Figure 2). A subsequent arteriogram showed new-onset active contrast extravasation from the esophageal branch of the left gastric artery. Gelfoam embolization was performed with complete cessation of extravasation.

**Figure 3:**
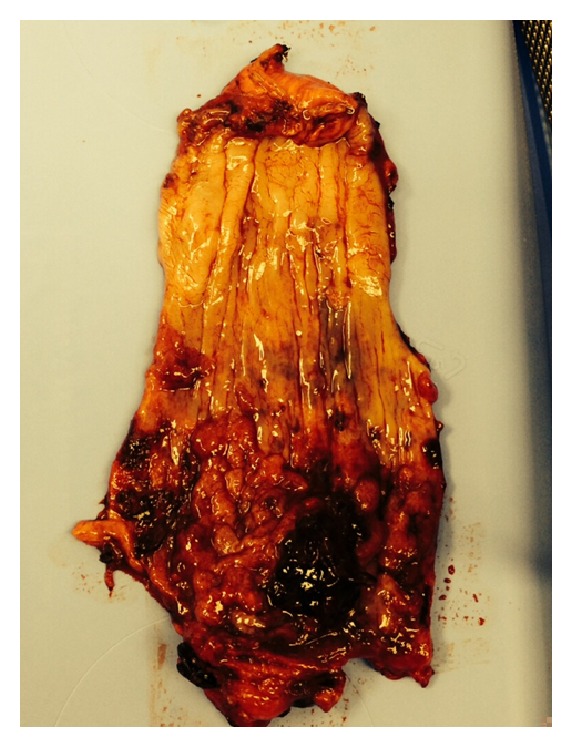
Submucosal hemorrhages and ulceration of the esophagus.

She eventually underwent surgical resection of a necrotic esophagogastric junction and thoracic esophagectomy with bipolar exclusion for recurrent bleeding about 2 weeks later. A cervical esophagostomy was created at the level of the left clavicle. Pathological examination demonstrated submucosal hemorrhage, esophagitis with dysplastic Barrett's mucosa, and an ulcer containing cytomegaloviral inclusions (Figure 3).

We report the first case of arterial bleeding from periesophageal pseudoaneurysms as well as use of embolization for arterial bleeding in the esophagus.

## 2. Discussion

Upper gastrointestinal bleeding from the esophagus is a common emergent condition in gastroenterology and often presents with hematemesis. Variceal bleeding and esophagitis are common causes of upper GI bleeding while vascular lesions are a relatively uncommon cause [1, 2]. Bleeding from periesophageal pseudoaneurysms, however, has not been reported in the literature.

The exact etiology of pseudoaneurysms in our patient is not clear but we speculate that vasculitis from SLE and recurrent cytomegalovirus may have contributed in its formation. We found only 2 case reports of SLE related pseudoaneurysms of the abdomen in which pseudoaneurysms developed as a sequel to pancreatitis [3, 4]. Thoracic pseudoaneurysms reported in literature are often associated with local trauma from a perforating bone [5, 6], injection of a toxin [7], or placement of a stent [8]. These are often present in the aorta or larger blood vessels. We did not find reports of pseudoaneurysms in the smaller periesophageal arterioles.

While endoscopy is the mainstay for the diagnosis and management of patients with upper gastrointestinal bleeding, a small cohort may fail endoscopic therapy. Transcatheter embolotherapy is an increasingly common intervention being performed for such patients [9].

Transarterial embolization in the esophagus for nonvariceal bleeding is a very rare intervention with only 5 cases of ulcer related bleeding reported in the literature [10]. There are also reports of embolization for bleeding in patients with esophageal cancer [11]. There is no literature on transarterial embolization of periesophageal pseudoaneurysms. While transcatheter arterial embolization was successful in our patient, definitive surgical intervention was still required for the management of esophageal necrosis. The embolization itself is likely to have contributed to the necrosis of the esophagogastric junction.

We present a rare case of bleeding from periesophageal pseudoaneurysms which were treated with transcatheter arterial embolization. To our knowledge, this entity as well as embolization as a management option has not been described before.
